# Power and sample-size calculations for trials that compare slopes over time: Introducing the slopepower command

**DOI:** 10.1177/1536867X211045512

**Published:** 2021-10-04

**Authors:** Stephen Nash, Katy E. Morgan, Chris Frost, Amy Mulick

**Affiliations:** Department of Infectious Disease Epidemiology London School of Hygiene and Tropical Medicine London, UK; Department of Medical Statistics London School of Hygiene and Tropical Medicine London, UK; Department of Medical Statistics London School of Hygiene and Tropical Medicine London, UK; Department of Non-communicable Disease Epidemiology London School of Hygiene and Tropical Medicine London, UK

**Keywords:** st0647, slopepower, power, sample-size calculations, slopes, parallel-arm trial

## Abstract

Trials of interventions that aim to slow disease progression may analyze a continuous outcome by comparing its change over time—its slope—between the treated and the untreated group using a linear mixed model. To perform a sample-size calculation for such a trial, one must have estimates of the parameters that govern the between- and within-subject variability in the outcome, which are often unknown. The algebra needed for the sample-size calculation can also be complex for such trial designs. We have written a new user-friendly command, slopepower, that performs sample-size or power calculations for trials that compare slope outcomes. The package is based on linear mixed-model methodology, described for this setting by Frost, Kenward, and Fox (2008, Statistics in Medicine 27: 3717–3731). In the first stage of this approach, slopepower obtains estimates of mean slopes together with variances and covariances from a linear mixed model fit to previously collected user-supplied data. In the second stage, these estimates are combined with user input about the target effectiveness of the treatment and design of the future trial to give an estimate of either a sample size or a statistical power. In this article, we present the slopepower command, briefly explain the methodology behind it, and demonstrate how it can be used to help plan a trial and compare the sample sizes needed for different trial designs.

## 1 Introduction

Sample size is a critical design consideration when planning a randomized controlled trial (RCT). Given an estimate of the target treatment effect, a formula for the variance of the treatment effect (which will depend on the trial design and analysis model), and the acceptable type I and type II error rates, the sample size is calculated with a simple algebraic formula (Campbell, Julious, and Altman 1995). However, for some designs and analysis models, the algebra to obtain the formula for the treatment-effect variance can be complex, and it can be difficult to derive reasonable guesses for the parameters that appear in that formula.

Consider a disease where progression can be measured by a continuous variable that is expected to deteriorate over time. Now consider an intervention whose aim is to slow that disease progression: we could use the continuous outcome as our trial outcome and see whether it responds to treatment over time. In such a trial, this outcome is typically recorded at participants’ baseline visits (prior to treatment allocation) and at least one follow-up visit with the aim of comparing randomized groups.

One way to analyze such an outcome is to use a linear mixed model (LMM) (Verbeke and Molenberghs 2000; Goldstein 2011; Longford 1993; Rabe-Hesketh and Skrondal 2012). In the simple case of a single follow-up measure and no missing data, a properly specified LMM can also be expressed as a generalized least-squares model (Frost, Kenward, and Fox 2008) and will give the same estimated treatment effect as analysis of covariance, albeit with reported standard errors that are only asymptotically equal (Frost, Kenward, and Fox 2008; White and Thompson 2005; Winkens et al. 2007). When there are multiple follow-up times, LMMs offer a flexible way of modeling the data that allows various assumptions to be made about the way the outcome changes over time. For example, it could be assumed that the outcome will change linearly over time in both groups and hence that the treatment difference between the groups is proportional to time (Frost, Kenward, and Fox 2008). LMMs also provide a convenient way of handling missing data, provided that a missing-at-random assumption can be made (Molenberghs and Kenward 2007).

Specifying the treatment-effect variance formula from such an LMM for a sample-size calculation requires knowledge of all the parameters that govern between- and within-subject variability in outcomes, which are often unknown. In such situations, one can use data from any relevant previously conducted longitudinal studies to estimate these parameters. We introduce a new package, slopepower, that translates a two-stage approach for estimating these parameters and performing sample-size calculations (Frost, Kenward, and Fox 2008; Frost et al. 2017) into a user-friendly command, appropriate for planning two-arm parallel trials comparing slopes where the treatment is expected to slow disease progression by a constant amount throughout follow-up and where the outcome is expected to change linearly over time.

slopepower estimates sample size by first fitting an LMM to a user-supplied longitudinal dataset and extracting estimates of slopes and components of between- and within-person variability. It then combines these estimates with other user inputs, including the number and spacing of the visits planned, to calculate the required sample size for a proposed RCT.

In section 2, we summarize existing methodology for estimating sample sizes for this design; in section 3, we describe the slopepower command; in section 4, we provide some examples of how to use slopepower; in section 4.2, we show how it can be used when planning a future trial; and in section 5, we give a short conclusion.

## 2 Methods

### 2.1 Future trial setup and analysis method

It is important to base a sample-size calculation on the model that will be used to analyze the trial. In this section, we therefore describe the sort of trial that slopepower could be applied to and the model that we assume will be used to analyze it. As described in section 1, we consider a parallel-arm trial in which the outcome of interest is a continuous measurement of disease that is expected to change over time and to respond to treatment. We consider that the outcome will be measured at a baseline visit and at least one follow-up visit, which occur at fixed time points for all participants.

When analyzing this outcome, we assume that it can be modeled as a linear change over time in the control group, with treatment acting to lessen that change proportionally over time. The analysis model can be written as

(1) $$y_{ij}=\beta_{0}+\beta_{1}t_{j}+\beta_{2}g_{i}t_{j}+a_{i}+b_{i}t_{j}+\varepsilon_{ij}$$

where *y_ij_* is the outcome for person i at time point *j*, *β*_0_ is the expected mean baseline measurement of the outcome in both arms, *β*_1_ is the change in the outcome over time (slope) in the control group, *t_j_* represents the times of the visits, *β*_2_ is the treatment effect (that is, the difference in slopes between the arms), *g_i_* is an indicator that is 0 in the control group and 1 in the active group for the *i*th person, *a_i_* is a random person-level intercept, *b_i_* is a random person-level slope, and $\varepsilon_{ij}∼N\left[0,\sigma_{\varepsilon }^{2}\right]$ is a normally distributed random-error term. The person-level random effects are assumed to be distributed as follows:

$$\left(\begin{array}{l} a_{i}\\ b_{i} \end{array}\right)∼N\left[\left(\begin{array}{l} 0\\ 0 \end{array}\right),\left(\begin{array}{cc} \sigma_{a}^{2} & \sigma_{ab}\\ \sigma_{ab} & \sigma_{b}^{2} \end{array}\right)\right]$$

Note that in this model, the baseline measure of the outcome, *y*_*i*0_, is treated as a correlated outcome. We assume that randomization is successful, so there is no expectation of a difference between the two groups at baseline (that is, at *t*_0_ = 0), and we estimate a single intercept for both arms. After baseline, it is assumed that the outcome changes linearly over time and that the treatment effect is therefore also constant and linear over time. In this formulation, the treatment effect is defined as the difference between the slope in the treated arm compared with that in the untreated arm.

Once the analysis model is specified, the treatment effect and its variance and thus sample-size requirements follow from the theory of linear mixed models (Frost, Kenward,and Fox 2008). A general formulation for a linear mixed model is

(2) Y∣u∼N(Xβ+Zu;R)foru∼N(0;G)

where **Y** is the vector of outcome variables, **X** is the design matrix, **β** is the vector of fixed effects, **Z** is the design matrix for the random effects, and **u** is the vector of random effects that are assumed to be distributed multivariate normally with mean **0** and covariance matrix **G** (note that this **G** is a matrix and should not be confused with the group indicator *g_i_*). Conditionally on the random effects, **Y** is assumed to have covariance matrix **R**.

Now, let us rewrite our model in a form that is not conditional on the random effects **u**. Marginally, (1) implies that

(3) $$Y∼N(X\beta ;\Sigma )\;\text{where}\;\Sigma =R+\text{ZG}{\text{Z}}^{T}$$

Here **Σ** is the variance–covariance matrix for unconditional **Y** and can be found from **R**, **Z**, and **G**. Provided that there is a postulated fixed value for the variance–covariance matrix, then

(4) $$\hat{\beta }={\left(X^{T}{\text{Σ}}^{-1}X\right)}^{-1}X^{T}\Sigma^{-1}Y$$

and

(5) $$V(\hat{\beta })={\left(X^{T}\Sigma^{-1}X\right)}^{-1}$$

Equation (4) can be used to estimate the treatment effect, while (5) defines a variance–covariance matrix for the estimated fixed parameters that permits calculation of the standard error of the treatment effect.

To illustrate these equations, let us relate (1) to our particular analysis model in (1) for the simple case of a two-person trial (one person per treatment group) with a baseline visit and two follow-up visits. In this case, we see that

$$Y=\left(\begin{array}{l} y_{10}\\ y_{11}\\ y_{12}\\ y_{20}\\ y_{21}\\ y_{22} \end{array}\right);\;X=\left(\begin{array}{ccc} 1 & 0 & 0\\ 1 & t_{1} & 0\\ 1 & t_{2} & 0\\ 1 & 0 & 0\\ 1 & t_{1} & t_{1}\\ 1 & t_{2} & t_{2} \end{array}\right);\;\beta =\left(\begin{array}{c} \beta_{0}\\ \beta_{1}\\ \beta_{2} \end{array}\right);\;Z=\left(\begin{array}{cccc} 1 & 0 & 0 & 0\\ 1 & t_{1} & 0 & 0\\ 1 & t_{2} & 0 & 0\\ 0 & 0 & 1 & 0\\ 0 & 0 & 1 & t_{1}\\ 0 & 0 & 1 & t_{2} \end{array}\right)$$

$$u=\left(\begin{array}{l} a_{1}\\ b_{1}\\ a_{2}\\ b_{2} \end{array}\right);\;R=\left(\begin{array}{cccccc} \sigma_{e}^{2} & 0 & 0 & 0 & 0 & 0\\ 0 & \sigma_{e}^{2} & 0 & 0 & 0 & 0\\ 0 & 0 & \sigma_{e}^{2} & 0 & 0 & 0\\ 0 & 0 & 0 & \sigma_{e}^{2} & 0 & 0\\ 0 & 0 & 0 & 0 & \sigma_{e}^{2} & 0\\ 0 & 0 & 0 & 0 & 0 & \sigma_{e}^{2} \end{array}\right);\;G=\left(\begin{array}{cccc} \sigma_{a}^{2} & \sigma_{ab} & 0 & 0\\ \sigma_{ab} & \sigma_{b}^{2} & 0 & 0\\ 0 & 0 & \sigma_{a}^{2} & \sigma_{ab}\\ 0 & 0 & \sigma_{ab} & \sigma_{b}^{2} \end{array}\right)$$

**Σ** from (3) therefore becomes a 6 × 6 matrix of form

$$\left(\begin{array}{cc} {\text{Σ}}^{*} & 0\\ 0 & {\text{Σ}}^{*} \end{array}\right)$$

where **0** is a 3 ×3 matrix of 0s and

$$\Sigma^{*}=\left(\begin{array}{ccc} \sigma_{a}^{2}+\sigma_{e}^{2} & \sigma_{a}^{2}+t_{1}\sigma_{ab} & \sigma_{a}^{2}+t_{2}\sigma_{ab}\\ \sigma_{a}^{2}+t_{1}\sigma_{ab} & \sigma_{a}^{2}+2t_{1}\sigma_{ab}+t_{1}^{2}\sigma_{b}^{2}+\sigma_{e}^{2} & \sigma_{a}^{2}+\left(t_{1}+t_{2}\right)\sigma_{ab}+t_{1}t_{2}\sigma_{b}^{2}\\ \sigma_{a}^{2}+t_{2}\sigma_{ab} & \sigma_{a}^{2}+\left(t_{1}+t_{2}\right)\sigma_{ab}+t_{1}t_{2}\sigma_{b}^{2} & \sigma_{a}^{2}+2t_{2}\sigma_{ab}+t_{2}^{2}\sigma_{b}^{2}+\sigma_{e}^{2} \end{array}\right)$$

and we can see that the algebra to obtain $V(\hat{\beta })$ from (5) is already fairly complex, even for this simple example. slopepower can perform the matrix calculations necessary to obtain $V(\hat{\beta })$ and hence the standard error for the estimated treatment effect, as we shall see in the next section.

### 2.2 Predicting a sample size for a future trial

Now that we have set up our trial design and analysis model, we can move on to how we would calculate a sample size for such a trial. For a sample-size calculation, we need a formula for the variance of the treatment-effect estimate, and we have shown how we can calculate this in the previous section. Because the matrices in (5) can get very large, we will use a simplifying trick—we shall first calculate the treatment-effect standard error for a two-person trial s*. Because the standard error for the treatment effect from a trial with N independent subjects in each arm is $s^{*}/\sqrt{N}$, it follows from standard theory that the sample size required to identify a postulated treatment difference *β*_2_ with statistical power *1* − *β* and two-sided significance level *α* is

(6) $$N={\left\{\frac{\left(z_{1-\alpha /2}+z_{1-\beta }\right)s^{*}}{\beta_{2}}\right\}}^{2}$$

Note that s* will depend on the design matrix **X** (which is itself dependent upon the number and spacing of the trial visits) and the variances and covariances from **R** and **G** ($\sigma_{e}^{2},\sigma_{a}^{2},\sigma_{b}^{2}$, and *σ_ab_*). Generally, appropriate values for these variances and covariances will not be known a priori, but estimates for these quantities can be obtained by fitting an appropriate linear mixed model to a previously collected dataset.

slopepower therefore estimates sample size in a two-stage process. In the first, it fits a linear mixed model to a user-supplied longitudinal dataset and extracts estimates of slopes and components of between- and within-person variability. In the second, it combines these estimates with other user inputs to calculate the required sample size for a proposed RCT, based on the analysis model given above in (1) and the sample-size formula in (6).

### 2.3 Stage 1: Slope and variance parameter estimation

slopepower uses the mixed command with the restricted maximum-likelihood (reml) option to fit a linear mixed model relating the outcome to time since study entry, using data supplied by the user. The data could be one of three different types:

1. Single group: dataset contains data from subjects with the disease of interest who are considered to be similar to the control group in the prospective trial. These may be subjects who are not receiving any treatment, for example, or are receiving standard of care. For simplicity, we shall refer to these subjects as untreated subjects. Such data could be from an observational study or from the control arm of a previously conducted RCT.
2. Two group, observational: optionally, the data can also include subjects without the disease (healthy controls).
3. Two group, RCT: again optionally, the data can include subjects with the disease who are receiving an additional treatment, possibly the treatment of interest in the future RCT (treated subjects).

First, let us consider a single-group dataset that contains only untreated subjects with the disease (situation 1 above). The outcomes *y_ij_* for person *i* at occasion *j* are modeled as a linear function of time elapsed since baseline *t_ij_* with random intercepts *a_i_*, slopes *b_i_*, and residual errors *ε_ij_*:

(7) $$\begin{array}{l} y_{ij}=\beta_{0}^{\prime }+\beta_{1}^{\prime }t_{ij}+a_{i}+b_{i}t_{ij}+\varepsilon_{ij}\\ \left(\begin{array}{l} a_{i}\\ b_{i} \end{array}\right)∼N\left[\left(\begin{array}{l} 0\\ 0 \end{array}\right),\left(\begin{array}{cc} \sigma_{a}^{2} & \sigma_{ab}\\ \sigma_{ab} & \sigma_{b}^{2} \end{array}\right)\right],\varepsilon_{ij}∼N\left(0,\sigma_{\varepsilon }^{2}\right) \end{array}$$

Note that we have marked the coefficients from the model in (7) with primes to distinguish them from the coefficients in the proposed analysis model for the future RCT from (1). Note also that time is now indexed by i and j because if the data are from an observational study, then visit times might vary by participant. For each person, the baseline visit is at time zero: *t*_*i*0_ = 0, and slopepower will rescale the times in the dataset if this is not the case.

The expected slope from the user-supplied data in (7) is $\beta_{1}^{\prime }$. This describes the expected change in the outcome per unit of time that would be seen without treatment in a person who has the disease under study. In this first stage, slopepower simply collects and stores the parameters from the model: $\beta_{1}^{\prime },\sigma_{a}^{2},\sigma_{b}^{2},\sigma_{ab}$, and $\sigma_{e}^{2}$ which will be used in stage 2 calculations.

If the supplied dataset also includes healthy controls (two-group, observational data, situation 2), then parameters are estimated separately in each group, such that the healthy controls have their own intercept, slope over time, and variances and covariances. It is possible to have slopepower run this model leaving out the random slopes over time for healthy controls (that is, neglecting $\sigma_{b}^{2}$ and *σ_ab_* for healthy controls) because the variability over time in healthy controls can sometimes be very small, leading to convergence issues in a model that tries to estimate these parameters.

Under this scenario, slopepower will store the slope $\left(\beta_{1,us}^{\prime }\right)$, variances, and covariances $\left(\sigma_{a}^{2},\sigma_{b}^{2},\sigma_{ab},\sigma_{\varepsilon }^{2}\right)$ from the untreated subjects. It will also store only the slope $\left(\beta_{1,hc}^{\prime }\right)$ from the healthy control group.

Finally, if the dataset is from a previous RCT and includes treated subjects (two-group, RCT data, situation 3), then the model in (1) is used. In this model, both groups have the same intercept because we expect the two groups to have the same mean at baseline under randomization, but the slopes over time are allowed to differ. The variance parameters are constrained to be the same in the two groups.

In this final scenario, slopepower will store the difference between the slopes in the treated and untreated groups $\left(\beta_{2}^{\prime }\right)$ and the joint variances and covariances $(\sigma_{a}^{2},\sigma_{b}^{2},\sigma_{ab},\sigma_{\varepsilon }^{2})$.

### 2.4 Stage 2: Treatment-effect variance estimation, sample-size calculation

In the second stage, slopepower assumes the trial under consideration will be analyzed using the model in (1). slopepower builds **Σ** for a two-person trial using (3) and the estimated variance and covariance parameters obtained from the first stage $(\sigma_{a}^{2},\sigma_{b}^{2},\sigma_{ab},\sigma_{\varepsilon }^{2})$. It then calculates the standard error of the treatment effect for this two-person trial, s*, by using (5). s* depends on the design matrix **X**, which is specified by the user, who tells slopepower the number and spacing of the visits for the future trial. Once s*is obtained, (6) is used to calculate the sample size.

In addition to s*, (6) depends on the target treatment effect. The command allows three scenarios regarding the effectiveness of the treatment under study. In these scenarios, the treatment effect is defined as being the following:

1. Toward no annual change; that is, it will reduce the rate of (future) change by a certain proportion of the way to zero. Under this scenario, a 100% effective treatment is defined as one that would halt change but not reverse it. Using singlegroup data without healthy controls or trial data from unrelated interventions implies this scenario. In this situation, the target treatment effect used in the sample-size calculation, *β*_2_, is calculated from the slope obtained from the user-supplied data $\left(\beta_{1}^{\prime }\right)$ and the user-supplied effectiveness, which we shall denote as e and which takes a value between 0 and 1:
  $$\beta_{2}=e\times \beta_{1}^{\prime }$$
2. Toward the slope observed in healthy controls; that is, it will reduce the rate of change over and above that seen in a disease-free population (the “excess” rate of change) by a certain proportion. Under this scenario, a 100% effective treatment would slow the change in subjects with the disease to the change observed in healthy controls but would not halt or reverse it. Using two-group observational data that include healthy controls implies this scenario, and the target treatment effect in this case is calculated as
  $$\beta_{2}=e\times \left(\beta_{1,us}^{\prime }-\beta_{1,hc}^{\prime }\right)$$
  where $\beta_{1,us}^{\prime }$ is the slope of the untreated subjects and $\beta_{1,hc}^{\prime }$ is the slope of the healthy controls obtained from the user-specified data.
  Note that the slope in the healthy controls could be interpreted as an upper limit on what is achievable with treatment, particularly when the outcome is expected to change over time even in healthy people. For example, say the outcome is a measure of cognitive decline in patients with Huntington’s disease (HD), and we know that even healthy people experience cognitive decline because of aging. Then, even a very effective treatment for patients with HD is unlikely to eliminate or reduce cognitive decline to a level below that of aging.
3. Equal to a previously observed treatment effect. For example, if a dataset from a previously conducted trial of the same or a similar treatment is available (perhaps a phase II trial that is being used to plan a phase III trial), the treatment effect observed in the previous trial can be used. Using such trial data, along with the appropriate usetrt option (described in section 3.2.2), implies this scenario. In this case, the target treatment effect is calculated as
  $$\beta_{2}=\beta_{2}^{'}$$
  where $\beta_{2}^{\prime }$ is the difference in slopes between the treatment and control arms from the model fit to the user-supplied RCT dataset.
  Note that one can also use a treatment effect that is proportional to the previously observed treatment effect in the previously conducted trial. This can be done by running the model under treatment effectiveness scenario 3 to obtain the sample size when targeting the previously observed treatment effect and then multiplying by the appropriate inflation factor (see example in section 4.1.3).

slopepower will use the user-supplied effectiveness (or the previously observed treatment effect, if specified) to calculate the target treatment effect for the future trial. It will then combine this with s* to calculate either the sample size or the power using (6).

The sample size calculated by slopepower thus depends upon the design matrix **X** (which is itself dependent upon the number and spacing of the trial visits) as well as the various components of variance and covariance that were estimated from the user-supplied data. These are assumed to be equal to what would be seen in a future trial setting.

Note that by fitting a model to data observed at discrete time points, slopepower can estimate the variance of the treatment effect for designs incorporating visits at any time points, including ones not in the original study. Trialists can use this flexibility to explore the sample-size implications for a range of designs that differ in length, number and timing of interim visits, and dropout patterns. We illustrate this in section 4.2.

### 2.5 Sample-size adjustment for trial dropouts

To compensate for individuals who withdraw early from the trial, slopepower can optionally adjust the required sample size using a pattern-mixture approach as advocated by Dawson and Lagakos (1991, 1993) and described by (Frost, Kenward, and Fox 2008). This approach is (appropriately) less conservative than upscaling the estimated sample size according to the anticipated proportion of individuals who reach the final visit. This is appropriate when interim data will be used to estimate the treatment effect, as is the case when using a mixed model such as that in (1) to analyze the trial.

In brief, slopepower assumes that individuals will be separated into strata according to dropout patterns. The approach first estimates for each such stratum the necessary sample size in the hypothetical situation that all individuals are in that stratum. The overall sample size is computed as the reciprocal of the weighted mean of the reciprocals of these strata-specific sample sizes, with the weights equal to the proportions of individuals anticipated to have each missing data pattern. Note that slopepower allows for missing data due to trial dropout but not for other patterns such as missed visits that result in intermittent missing values during follow-up.

### 2.6 Some notes of caution

It is important that the dataset used for the first stage of model fitting is from a population that is sufficiently similar to that in the proposed trial so that we can generalize the estimates of the variance parameters to the planned RCT. In practice, that might mean that inclusion criteria used in the previous dataset are similar to those proposed in the future trial and that the untreated subjects suffer from a severity of disease similar to that expected in the participants of the planned trial at baseline. It may be that no such dataset exists, and in such a case it might be necessary to collect some data in a pilot study.

Note that, as always, variances and covariances will be estimated more precisely given more people and time points in the dataset. Users should proceed with caution, especially if they have a small dataset, and be aware that their sample-size estimates will contain uncertainty due to the estimation of the variance parameters in the first stage. slopepower can be run with Stata’s bootstrap prefix to obtain a 95% confidence interval (CI) for a sample-size estimate, although the user should be sure to account for the structure of the data when doing so. Care should also be taken when bootstrapping small datasets, particularly those with outlying values, because the coverage of a bootstrap CI might then not be close to its nominal value. In addition, if the estimated slope in the user-supplied dataset is not large relative to its standard error (as a rule of thumb, we recommend that the ratio of the magnitude of the estimate to its slope should be greater than 2.5), the bootstrap samples may possibly yield estimates of the mean slope that are both negative and positive, meaning that some of the bootstrap samples will relate to trials that are trying to reduce a positive slope while others will relate to trials that are trying to reduce the magnitude of a negative slope, hence rendering the CI meaningless. However, such cases should be unlikely because if a trial is being contemplated to reduce a slope, then there should be strong evidence of a trend over time such that the estimated slope is substantially larger than its standard error in the user-supplied dataset. An example of how bootstrap can be used with slopepower is given in section 4.1.2.

As with any statistical model, one can make out-of-sample predictions. The command slopepower does not give a warning when estimating sample sizes for trials of duration longer than the maximum length of follow-up observed in the given data, so users should be aware of the assumptions that are made when doing this. Similarly, slopepower interpolates between time points. This is a necessary assumption to make to be able to consider trial designs with visit spacing that differs from the original dataset, but users should be aware that it is assumed that the model holds across the time scale of interest.

Also note that, other than subject-specific random effects, slopepower has no capability to model dependency between observations, such as center- or visit-specific effects. This implies that, conditional on subject, observations are assumed to be independent.

## 3 The slopepower command

The syntax of slopepower is as follows:

slopepower *depvar*  [*if*]  [*in*], subject(*varname*) time(*varname*)

   schedule(*numlist*) { obs | rct} [nocontrols casecon(*varname*)

   treat(*varname*) dropouts(*numlist*) scale(#) alpha(#) power(#) n(#)

   [effectiveness(#) |usetrt] iterate(#) nocontvar]

### 3.1 Description

slopepower will calculate sample size or power for trials where the outcome is a slope, using a two-stage approach. In the first stage, slopepower uses data in memory (provided by the user) to estimate the necessary slopes, variances, and covariances. In the second stage, it uses these estimates, along with user-specified information, to calculate a sample size or power.

The user-provided dataset can be of three basic types as described in section 2.3: containing subjects with the disease who are untreated only (or minimally treated, for example, receiving standard of care); containing untreated subjects with the disease and healthy controls; or a previous RCT containing subjects who are untreated and subjects who are treated. In all cases, the data should contain repeated measurements of the outcome in long format (see reshape for more details). A linear mixed model is run (using mixed) on the data in memory to estimate the relevant parameters. The data in memory are not altered by slopepower.

### 3.2 Options

#### 3.2.1 Options for data in memory

subject(*varname*) is the unique identifier for participants in the user-supplied dataset. subject() is required.

time(*varname*) is the time variable of visits in the dataset. This can be in any units (for example, days, months, years). It is assumed to be time since start of observation for each individual. If it is not (for instance, if it is an actual calendar date), slopepower will issue a warning and rescale it accordingly. time() is required.

obs and rct tell Stata the nature of the data in memory. obs should generally be used for observational data and rct for previously collected trial data (with an exception mentioned below). Exactly one of obs or rct must be specified.

nocontrols should be used with obs if all the subjects in your observational data have the condition of interest (that is, if there are no healthy controls).

casecon(*varname*) specifies the variable used to identify cases in observational data; it can be used only with obs. It must be a binary 0/1 variable with cases coded as 1.

treat(*varname*) specifies the treatment variable when you are using RCT data; it can be specified only with rct. It must be a binary 0/1 variable with the experimental group coded as 1.

#### 3.2.2 Options for planned trial

schedule(*numlist*) specifies the visit times for the proposed trial. A baseline visit at time 0 is assumed; this list should describe subsequent visits in whole-number units of time. The default is to use the same time unit as the time variable in the dataset. To use a different timescale, specify how many time() units make one schedule() unit in the scale() option. schedule() is required.

dropouts(*numlist*) specifies the estimated proportion of dropouts you expect at each study visit. It must correspond exactly to the schedule list. Each number in the list is a proportion between 0 and 1; this is the fraction of subjects (of those who start the trial) you estimate will fail to attend that visit. We follow the pattern-mixture method of Dawson and Lagakos (1991, 1993) (see section 2.5).

scale(#) specifies the ratio between the time and visit timescales. For instance, if the time variable in your dataset is in days and you wish to have visits annually for three years, you would specify scale(365) and schedule(1 2 3).

alpha(#) sets the significance level (also known as type I error rate) to be used in the planned study. The default is alpha(0.05).

power(#) sets the power for the planned study. The default is power(0.8). This option is required to compute the sample size.

n(#) specifies the total number of participants who will be in the trial. If an odd number is given, *n* − *1* will be used to allow equal numbers per arm. This option is required to compute the power. Only one of power() or n() may be specified.

effectiveness(#) and usetrt specify the effect size you would like to be able to detect in the future trial. effectiveness() specifies this effect size as a proportion of the difference between cases and healthy controls in the observational data in memory. If RCT data, or observational data with no healthy controls, are used, effectiveness() is a proportion of the difference toward a slope of 0. This must be a number between 0 and 1; the default is effectiveness(0.25). usetrt specifies that, when RCT data are used, the planned study is targeting the same effect size as observed in the previous dataset. You can specify only one of effectiveness() or usetrt.

#### 3.2.3 Model options

iterate(#) is used as an option in the mixed command, which specifies the maximum number of iterations allowed in the mixed model.

nocontvar specifies that the mixed model should not estimate a random-slope variance parameter or the covariance between random slopes and intercepts for healthy controls. This is applicable only when you are using observational data with healthy controls. Ignoring this variance and covariance may help the model to converge.

## 4 Examples

### 4.1 How to use the code

In this section, we use simulated data to illustrate the options described above. The three examples given cover the three types of data that can be used with slopepower: single-group data (with only untreated subjects); two-group, observational data (with untreated subjects and healthy controls); and two-group, RCT data (treated and untreated subjects).

These example datasets together contain three groups of people:

1. people with HD who are receiving standard of care (untreated subjects);
2. people without HD (or the genetic mutation that leads to it) (healthy controls); and
3. people with HD who are being treated as part of a trial (treated subjects).

Section 4.1.1 describes the situation when you have a dataset containing only people from group 1. Section 4.1.2 describes a dataset containing people from groups 1 and 2, and section 4.1.3 is for a dataset containing groups 1 and 3.

In all datasets, we have assumed that the “cases” (or untreated subjects) are people with HD, a neurodegenerative disorder in which cognitive functioning typically declines during disease progression. The outcome of interest is their score on the Symbol Digits Modalities Test (Smith 2007), a measure of cognitive function taking integer values between 0 and 110, with higher scores indicating better function. We have not simulated any missing data. In all cases, the data are in long format, ready for use with slopepower.

#### 4.1.1 Single-group data with untreated subjects only

We have simulated three years of data on 200 people with HD, with measurements recorded each year; the visit variable indicates the year of follow-up. Values for sdmt are simulated according to the model in (7) using parameter values of $\beta_{0}^{\prime }=34,\;\;\beta_{1}^{\prime }=-1.8,\sigma_{a}^{2}=100,\sigma_{b}^{2}=2,\sigma_{ab}=5$, and $\sigma_{\varepsilon }^{2}=10$. Outcome values are then truncated at zero and rounded to the nearest integer. Code for generating the data is given in the appendix. Data for the first two participants are shown below:

**Table:** 

id	visit	sdmt
1	0	41
1	1	33
1	2	25
1	3	30
2	0	16
2	1	14
2	2	13
2	3	6

We first show the syntax to plan an RCT with annual visits over two years, assuming no dropouts, with 80% power to detect a treatment effect that will eliminate one-third of the slope. Note that here the assumed effectiveness is toward “no annual change” or a slope of zero. The obs option identifies the data in memory as being observational (although note that it would be possible to use a dataset containing only the untreated arm from an RCT with this option), and, with no healthy controls in the dataset, we use the nocontrols option. The default values of 5% type I error and 80% power are used.
. slopepower sdmt, schedule(1 2) subject(id) time(visit) obs nocontrols
> effectiveness(0.33)
Data characteristics:
               Number of observations in model = 800
                         Participants in model = 200
                                Slope of cases = -1.672
Parameters for planned study:
                                         alpha = 0.050
                                         power = 0.800
                                 effectiveness = 0.330
         target treatment difference in slopes = 0.552
                    number of follow-up visits = 2
                         schedule (and dropouts) : 1, 2
                                         scale = 1
Estimated sample size:
                                             N = 712
                                     N per arm = 356

This shows that a total sample size of 712 will be required for the planned trial. The first section of the output shows three results from the linear model run on the data in memory: the number of observations and subjects that were included in the model and the estimated slope from the data. The remaining output confirms the user-contributed parameters, or the defaults used if they were not specified, and gives the target treatment effect that slopepower calculates from the model slope estimates and the user-inputted effectiveness: this is *β*_2_ in (6). Finally, slopepower gives the estimated sample size both as a total and per arm.

Visits do not have to be scheduled at regular intervals. If you wish to extend the above trial to five years, with no additional interim visits, you would specify the command below. However, note that this is extending the estimates out of the initial sample duration. Here we have also assumed that 10% of participants would be lost to follow-up between the visit at year two and the final visit.

. slopepower sdmt, schedule(1 2 5) subject(id) time(visit) obs nocontrols
> effectiveness(0.33) dropouts(0 0 0.1)
Data characteristics:
             Number of observations in model = 800
                       Participants in model = 200
                              Slope of cases = -1.672
Parameters for planned study:
                                       alpha = 0.050
                                       power = 0.800
                               effectiveness = 0.330
       target treatment difference in slopes = 0.552
                  number of follow-up visits = 3
                     schedule (and dropouts) : 1 (0), 2 (0), 5 (0.1)
                                       scale = 1
Estimated sample size:
                                           N = 328
                                   N per arm = 164

Here the sample size is reduced because of the extended follow-up, despite the loss to follow-up, which is shown as a proportion in parentheses after each visit in the schedule list.

If you wish to schedule visits every six months, you must use the scale() option to indicate that half a unit in the observed timescale is equivalent to one unit in the RCT timescale. Hence, the timescale specified in the command below is in increments of six months, and the trial is scheduled to last two years.

. slopepower sdmt, schedule(1 2 3 4) scale(0.5) subject(id) time(visit)
> obs nocontrols effectiveness(0.33)
Data characteristics:
                              Number of observations in model = 800
                                        Participants in model = 200
                                               Slope of cases = -0.836
Parameters for planned study:
                                                        alpha = 0.050
                                                        power = 0.800
                                                effectiveness = 0.330
                        target treatment difference in slopes = 0.276
                                   number of follow-up visits = 4
                                      schedule (and dropouts) : 1, 2, 3, 4
                                                        scale =.5
Estimated sample size:
                                                            N = 620
                                                    N per arm = 310

Again, the sample size is slightly reduced compared with the first example because of an increase in efficiency gained from the interim visits. Also note that the slope observed in the data has halved; this is because it is reported in the units of the planned trial, so here it relates to a difference per six months (rather than per year as in the earlier examples).

#### 4.1.2 Observational data with cases and healthy controls

Here we have simulated 250 people with HD and 250 without, with dates of observation used rather than visit number. For cases (or untreated subjects), the sdmt score was generated as above. For controls, we assumed a mean at baseline of 53 and an increasing average annual change (due to a practice effect) of 0.9. Variance and covariance parameters of $\sigma_{a}^{2}=75,\sigma_{b}^{2}=1,\sigma_{ab}=1$, and $\sigma_{\varepsilon }^{2}=10$ were used for healthy controls. Data for the first control and the first case are shown below:

**Table:** 

id		case	vdate	sdmt
1	Healthy	control	11jul2009	40
1	Healthy	control	21jun2010	46
1	Healthy	control	06jul2011	41
1	Healthy	control	06may2012	45
251		Case	06jun2009	35
251		Case	13aug2010	34
251		Case	31aug2011	36
251		Case	05sep2012	39

Note that case is a labeled numeric variable and takes value 0 for healthy controls and 1 for cases.

Because we now have healthy controls in our data, we drop the nocontrols option and instead use casecon() to tell slopepower which variable identifies the untreated subjects in our dataset. Because the time variable is a date (recorded in days) and we wish to specify our RCT schedule in years, we use the scale() option.
. slopepower sdmt, schedule(1 2) scale(365) subject(id) time(vdate)
>	obs casecon(case) effectiveness(0.33)
WARNING: time variable did not start at zero for all participants. Times have
>	been adjusted such that the first visit for each person is treated as time
>	zero.
Data characteristics:
                  number of observations in model = 2000
                  number of participants in model = 500
                    observed difference in slopes = -2.690
                                   slope of cases = -1.715
                        slope of healthy controls = 0.975
Parameters for planned study:
                                            alpha = 0.050
                                            power = 0.800
                                    effectiveness = 0.330
            target treatment difference in slopes = 0.888
                       number of follow-up visits = 2
                          schedule (and dropouts) : 1, 2
                                            scale = 365
Estimated sample size:
                                                N = 296
                                        N per arm = 148

The first thing to note is that because the time variable is a date, slopepower has issued a warning to let you know that it has been transformed in the model so that the earliest date for each individual is at time zero—this is necessary to ensure the intercept is estimated at baseline and that the covariance between the random slopes and intercepts is correctly estimated. Note that now there are two slopes reported in the output—one for the cases and one for the healthy controls. The effectiveness is now applied to the difference between these two slopes, which is also provided in the output.

The output shows that a total sample size of 296 will be required for the planned trial. The decreased sample size compared with that in the previous section is partly because here we have an estimate for the slope of healthy individuals, so instead of relating our effectiveness to no change over time (a slope of zero), we relate it to the difference between the slope in untreated subjects and that in healthy controls. Hence, the target treatment effect is larger here than above, even though an effectiveness of 0.33 was specified both times, because the healthy controls have a positive slope.

Let us suppose that we are interested in obtaining a bias-corrected and accelerated bootstrap CI for this predicted sample size. We can do this by using the following command: . bootstrap r(sampsize), cluster(id) idcluster(id2) strata(case) rep(2000)
> seed(123) bca jack(n(r(obs_in_model))): slopepower sdmt, schedule(1 2)
> scale(365) subject(id) time(vdate) obs case(case) effectiveness(0.33)
WARNING: time variable did not start at zero for all participants. Times have
> been adjusted such that the first visit for each person is treated as time zero.
warning: Because slopepower is not an estimation command or does not set
	 e(sample), bootstrap has no way to determine which observations are
	 used in calculating the statistics and so assumes that all
	 observations are used. This means that no observations will be
	 excluded from the resampling because of missing values or other
	 reasons.
	 If the assumption is not true, press Break, save the data, and drop
	 the observations that are to be excluded. Be sure that the dataset in
	 memory contains only the relevant data.
(running slopepower on estimation sample)
WARNING: time variable did not start at zero for all participants. Times have
> been adjusted such that the first visit for each person is treated as time zero.
                                           
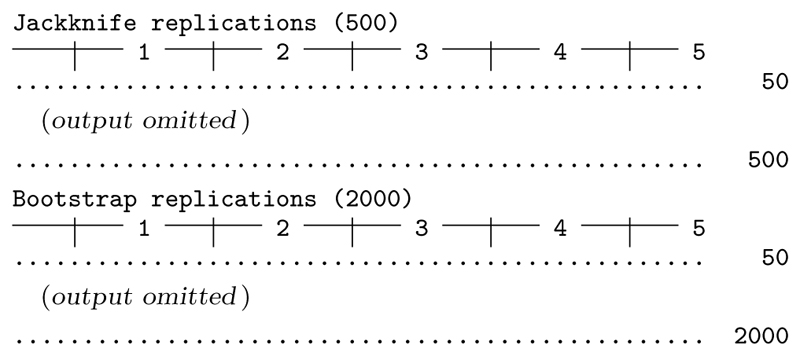

Bootstrap results
Number of strata     =		 2 		Number of obs  =  2,000
						Replications   =  2,000
	command: slopepower sdmt, schedule(1 2) scale(365) subject(id)
		    time(vdate) obs case(case) effectiveness(0.33)
	  _bs_1: r(sampsize)

**Table:** 

(Replications based on 500 clusters in id)						
	Observed	Bootstrap			Normal-based	
	Coef.	Std. Err.	z	P>|z|	[95% Conf. Interval]	
_bs_1	296	30.0739	9.84	0.000	237.0562	354.9438



              . estat bootstrap, bca
Bootstrap results
Number of strata     =    	2 		Number of obs  =  2,000
						Replications   =   2000
	command: slopepower sdmt, schedule(1 2) scale(365) subject(id)
		    time(vdate) obs case(case) effectiveness(0.33)
	  _bs_1: r(sampsize)
	  
            


**Table:** 

(Replications based on 500 clusters in id)						
	Observed		Bootstrap		
	Coef.	Bias	Std. Err.	[95% Conf. Interval]	
_bs_1	296	-17.738	30.073902	262	402 (BCa)
(BCa) bias-corrected and accelerated confidence interval					

There are several important things to note about the bootstrap command. First, we have specified that we require a bootstrap CI for the sample size, which is saved by slopepower as r(sampsize). Second, we need to use the cluster() option so that people, rather than individual data points, are sampled from the dataset. We also need to use the idcluster() option, with a new identifier called id2, so that if one person appears twice in a bootstrap sample, he or she is treated as two separate people rather than as one person with twice as many data points than as in the data itself. Third, we need the option strata() so that cases and healthy controls are sampled separately. Finally, because we want a bias-corrected and accelerated CI, we have to tell Stata where slopepower saves the number of observations in each model using the jack(n(r(obs_in_model))) option. Opting for a bias-corrected and accelerated CI is recommended because the distribution of estimated sample sizes across the bootstrap samples is likely to be skewed. We can see in this example that the CI extends substantially further above the central value (up to a sample size of 402) than it does below it (down to a sample size of 262).

One can also calculate the power for a specified sample size by using the n() option instead of power(). Note that this n() refers to the total sample size and assumes a 1:1 ratio between the two treatment groups. Here we also assume a dropout rate of 5% per year of those who start the trial.


. slopepower sdmt, schedule(1 2) scale(365) subject(id) time(vdate)
> obs case(case) effectiveness(0.33) n(200) dropouts(0.05 0.05)
WARNING: time variable did not start at zero for all participants. Times have
> been adjusted such that the first visit for each person is treated as time zero.
Data characteristics:
          number of observations in model = 2000
          number of participants in model = 500
            observed difference in slopes = -2.690
                           slope of cases = -1.715
                slope of healthy controls = 0.975
Parameters for planned study:
                                    alpha = 0.050
                              specified N = 200
                                 actual N = 200
                                N per arm = 100
                            effectiveness = 0.330
    target treatment difference in slopes = 0.888
               number of follow-up visits = 2
                  schedule (and dropouts) = 1 (0.05), 2 (0.05)
                                    scale = 365
Estimated power:
                                    power = 0.597


The estimated power is 60%. The other main difference in output here is that two values for the total *N* are given: the value specified by the user and the value actually used in the power calculation, which is either *n* or *n* − 1 if the user specified an odd number.

#### 4.1.3 RCT data with treated and untreated groups

The simulated RCT data contain 75 people who received treatment and 75 who did not receive active treatment. In this dataset, the outcome was generated from a model with an intercept of 34, a slope in the untreated arm of −1.8 units/year, a slope in the treated arm of −0.8 units/year, and variance and covariance parameters as in section 4.1.1.

Example data from one participant in each arm are shown here:

**Table:** 

id	treat	visit	sdmt
1	Placebo	0	35
1	Placebo	.5	36
1	Placebo	2	34
76	Treat	0	29
76	Treat	.5	33
76	Treat	2	35

Again, note that treat is a labeled numeric variable, where Placebo (untreated arm) takes value 0 and Treat (treated arm) takes value 1.

If the aim of the planned study is to detect the same effect size as in the previous RCT, then the usetrt option should be used. Here we show the syntax to produce a sample-size estimate for a three-year study with one interim visit at year two and loss to follow-up of 10% per year of those who start the trial. Note that we now use the rct option instead of obs.

. slopepower sdmt, schedule(2 3) subject(id) time(visit) rct treat(treat)
> usetrt dropout(0.2 0.1)
Data characteristics:
          number of observations in model = 450
          number of participants in model = 150
            observed difference in slopes = -0.747
                     slope of control arm = -1.852
                slope of experimental arm = -1.104
Parameters for planned study:
                                    alpha = 0.050
                                    power = 0.800
                            effectiveness =	.
    target treatment difference in slopes = 0.747
               number of follow-up visits = 2
                  schedule (and dropouts) : 2 (0.2), 3 (0.1)
                                    scale = 1
Estimated sample size:
                                        N = 318
                                N per arm = 159

Here we see that a sample size of 318 is required to detect a 0.75 units per year change in annual decline that was seen in the previous RCT.

Suppose that the previous RCT is a pilot study or phase II trial and that the investigators suspect that, because of its small size, the treatment effect might have been overestimated. They may wish to plan the future RCT such that it has power to detect a treatment effect that is 50% of that observed previously. To do this, we can multiply the sample size above by 4 (that is, 1 over 0.5 squared), so we would need a sample size of 1,272. More generally, note that if we want a sample size for a target treatment effect that is *p* times that observed in the previous trial, *N_p_*, we need to multiply the N that uses the previously observed treatment effect (318 in this example) by *p*^−2^. This follows from (6):

$$N_{p}={\left(\frac{\left(z_{1-\alpha /2}+z_{1-\beta }\right)s^{*}}{p\beta_{2}}\right)}^{2}=\frac{1}{p^{2}}{\left(\frac{\left(z_{1-\alpha /2}+z_{1-\beta }\right)s^{*}}{\beta_{2}}\right)}^{2}=\frac{1}{p^{2}}N$$

Note that if we had data from a previous RCT that was trialing a completely different treatment from that under consideration in the future trial, we might have decided to use only the untreated arm as our dataset and use the options for a single group of untreated subjects as shown in section 4.1.1.

### 4.2 Exploring future trial designs with slopepower

slopepower can be used to explore sample sizes under a variety of scenarios, which may be of use when planning the future trial. Here we suppose we are planning a three-year study, using the observational dataset described in section 4.1.1 and targeting the same 33% effectiveness. We assume that we will be able to recruit a total of 450 participants, and we report the estimated power for several different scenarios that explore different patterns of follow-up visits and dropouts. The code to obtain these results is given in the appendix.

As can be seen from table 1, adding extra follow-up visits increases the power. For example, when there are no dropouts, the power increases from around 80% with a single follow-up visit to almost 87% with six-month follow-up visits. As the anticipated rate of dropouts increases, the trial designs that include extra follow-up visits become increasingly efficient because they allow data collected at interim visits to be used in the analysis. Note that in this simulated example, when 10% of participants are expected to be lost each year, adding six-month visits recovers information to the extent that it achieves nearly the same power as a trial with a single follow-up visit with no dropouts.

## 5 Conclusion

We have presented a new command, slopepower, that can be used to perform samplesize or power calculations for trials that compare rates of change in an outcome (the slope) over time. slopepower can be used for any continuous clinical trial outcome that is expected to change at a constant rate over time and where a treatment is expected to slow that rate. This might include continuous outcomes such as log10-transformed total kidney volume (Torres et al. 2012), disease severity scores such as the Amyotrophic Lateral Sclerosis Functional Rating Scale-Revised (Paganoni et al. 2020), body mass index (Attia et al. 2019), carotid intima-media thickness (Kastelein et al. 2007), biomarkers such as log10-transformed C-reactive protein degraded by matrix metalloproteinases 1 and 8 (Maher et al. 2019), or variables measuring lung function such as forced expiratory volume (McCormack et al. 2011) or forced vital capacity (Raghu et al. 2018).

The package is based on linear mixed-model methodology, described for this setting by Frost, Kenward, and Fox (2008), and requires a user-supplied dataset containing longitudinal data on a similar population to that expected in the future trial. In the first stage of this approach, slopepower obtains estimates of the mean rate of change in the outcome, together with variances and covariances, from a linear mixed model fit to user-supplied data. In the second stage, these estimates are combined with user input on the target effectiveness of the treatment and design of the future trial to give an estimate of a sample size for, or the statistical power of, the future trial. This command provides, to our knowledge, for the first time a convenient way to calculate such estimates for trials with repeated measures that aim to alter rates of change in an outcome.

## Supplementary Material

Appendix

## Figures and Tables

**Table 1:** Estimated power for different trial designs and dropout scenarios

Planned trial design	Dropouts	Power
Baseline and final visit (three years) only	None	79.8%
Annual follow-up visits	None	81.7%
Six-month follow-up visits	None	86.5%
Baseline and final visit (three years) only	5% per year	73.2%
Annual follow-up visits	5% per year	77.1%
Six-month follow-up visits	5% per year	82.8%
Baseline and final visit (three years) only	10% per year	64.8%
Annual follow-up visits	10% per year	71.6%
Six-month follow-up visits	10% per year	78.3%

## 7 Programs and supplemental materials

To install a snapshot of the corresponding software files as they existed at the time of publication of this article, type

. net sj 21-3

. net install st0647 (to install program files, if available)

. net get st0647 (to install ancillary files, if available)
