# Supplementary material for: Power and sample-size calculations for trials that compare slopes over time: Introducing the slopepower command
Source: Stata J. Author manuscript; Available in PMC 2023 Jul 20. (PMC7614632; doi:10.1177/1536867X211045512)
Supplement: Appendix [file EMS163747-supplement-Appendix.pdf]

A Appendix

Here we provide the code used to simulate example datasets used in section 4. All data were generated using Stata 16.

```
* Data for example 4.1: single-group data, untreated subjects only
set seed 5221

* create dataset of 200 people
clear
set obs 200
generate id = _n
```

```
* draw random person intercepts and slopes
drawnorm a b, cov(100, 5 \ 5, 2)

* create more visits
expand 4
by id, sort: generate visit = _n - 1

* generate sdmt from model in (7)
generate sdmt = 34 -1.8 * visit + a + b * visit + rnormal(0, 10^0.5)
summarize sdmt

count if sdmt < 0

* truncate at zero, and round to nearest integer to create a score like sdmt
replace sdmt = round(sdmt)
replace sdmt = 0 if sdmt < 0

* drop person intercept and slope, which are no longer needed
drop a b

save slpower1, replace

* list of two ids from article
list if id == 1 | id == 2, separator(4) noobs

* slopepower commands from article
slopepower sdmt, schedule(1 2) subject(id) time(visit) obs nocontrols       ///
     effectiveness(0.33)

slopepower sdmt, schedule(1 2 5) subject(id) time(visit) obs nocontrols     ///
     effectiveness(0.33) dropouts(0 0 0.1)

slopepower sdmt, schedule(1 2 3 4) scale(0.5) subject(id) time(visit)       ///
     obs nocontrols effectiveness(0.33)


* Data for example 4.2: two-group data, observational, untreated subjects, and
* healthy controls
set seed 1146

* create dataset of 500 people, with 250 untreated subjects and 250 healthy
* controls
clear
set obs 500
generate id = _n
generate case = (_n > `=_N/2')

* draw two sets of random intercepts and slopes, and use a, b for cases and
* c, d for controls
drawnorm a b, cov(100, 5 \ 5, 2)
drawnorm c d, cov(75, 1 \ 1, 1)

* create more visits
expand 4
by id, sort: generate visit = _n - 1
```

```
* generate sdmt from model in (7)
generate sdmt = 34 - 1.8 * visit + a + b * visit + rnormal(0, 10^0.5) if case == 1
replace sdmt = 53 + 0.9 * visit + c + d * visit + rnormal(0, 10^0.5) if case == 0
summarize sdmt

count if sdmt < 0

* truncate at zero, and round to nearest integer to create a score like sdmt
replace sdmt = round(sdmt)
replace sdmt = 0 if sdmt < 0

* generate visit dates that vary for each individual
generate vdate = (visit * 365) + date("5 July 2009", "DMY")
replace vdate = vdate + int(rnormal(0, 50))
format vdate %td

* drop visit, person intercept, and slope, which are no longer needed
drop visit a b c d

* label case variable
label define case_lab 0 "Healthy control" 1 "Case"
label values case case_lab

order id case vdate sdmt

save slpower2, replace

* list of two ids from article
list if id == 1 | id == 251, separator(4) noobs

* slopepower commands from article
slopepower sdmt, schedule(1 2) scale(365) subject(id) time(vdate)          ///
     obs casecon(case) effectiveness(0.33)

bootstrap r(sampsize), cluster(id) idcluster(id2) strata(case) rep(2000)    ///
     seed(123) bca jack(n(r(obs_in_model))): slopepower sdmt, schedule(1 2) ///
     scale(365) subject(id) time(vdate) obs casecon(case) effectiveness(0.33)
estat bootstrap, bca

slopepower sdmt, schedule(1 2) scale(365) subject(id) time(vdate)          ///
     obs casecon(case) effectiveness(0.33) n(200) dropouts(0.05 0.05)


* Data for example 4.3: two-group, rct, untreated subjects (cases), and treated
* subjects
set seed 1021

* create dataset of 150 people, with 75 in each treatment arm
clear
set obs 150
generate id = _n
generate treat = (_n > `=_N/2')

* draw random person intercepts and slopes
drawnorm a b, cov(100, 5 \ 5, 2)
```

```
* create more visits
expand 3
by id, sort: generate visit = _n - 1
replace visit = 0.5 if visit == 1

* generate sdmt from model in (1)
generate sdmt = 34 - 1.8 * visit + 1 * treat * visit + a + b * visit +    ///
     rnormal(0, 10^0.5)

replace sdmt = round(sdmt)

* label treatment variable
label define treat_lab 0 "Placebo" 1 "Treat"
label values treat treat_lab


* drop person intercept and slope, which are no longer needed
drop a b

save slpower3, replace

* list of two ids from article
list if id == 1 | id == 76, separator(3) noobs

* slopepower commands from article
slopepower sdmt, schedule(2 3) subject(id) time(visit) rct treat(treat)   ///
     usetrt dropout(0.2 0.1)

***********************
* commands to produce table 1

* use same dataset as first example
use slpower1, clear

* no dropout
slopepower sdmt, schedule(3) subject(id) time(visit) obs nocontrols       ///
     effectiveness(0.33) n(450)
slopepower sdmt, schedule(1 2 3) subject(id) time(visit) obs nocontrols   ///
     effectiveness(0.33) n(450)
slopepower sdmt, schedule(1 2 3 4 5 6) scale(0.5) subject(id) time(visit) ///
     obs nocontrols effectiveness(0.33) n(450)

* 5% dropout per year = 15% over 3 years
slopepower sdmt, schedule(3) subject(id) time(visit) obs nocontrols       ///
     effectiveness(0.33) n(450) dropout(0.15)
slopepower sdmt, schedule(1 2 3) subject(id) time(visit) obs nocontrols   ///
     effectiveness(0.33) n(450) dropout(0.05 0.05 0.05)
slopepower sdmt, schedule(1 2 3 4 5 6) scale(0.5) subject(id) time(visit) ///
     obs nocontrols effectiveness(0.33) n(450)                            ///
     dropout(0.025 0.025 0.025 0.025 0.025 0.025)

* 10% dropout
slopepower sdmt, schedule(3) subject(id) time(visit) obs nocontrols       ///
     effectiveness(0.33) n(450) dropout(0.3)
slopepower sdmt, schedule(1 2 3) subject(id) time(visit) obs nocontrols   ///
     effectiveness(0.33) n(450) dropout(0.1 0.1 0.1)
slopepower sdmt, schedule(1 2 3 4 5 6) scale(0.5) subject(id) time(visit) ///
     obs nocontrols effectiveness(0.33) n(450)                            ///
     dropout(0.05 0.05 0.05 0.05 0.05 0.05)
```
